# Experimental, Numerical and Analytical Evaluation of Load-Bearing Capacity of Cold-Formed S-Beam with Sectional Transverse Strengthening

**DOI:** 10.3390/ma17246198

**Published:** 2024-12-18

**Authors:** Robert Studziński, Volodymyr Semko, Katarzyna Ciesielczyk, Mateusz Fabisiak

**Affiliations:** Faculty of Civil and Transport Engineering, Poznan University of Technology, Piotrowo 5 Street, 60-965 Poznan, Poland; volodymyr.semko@put.poznan.pl (V.S.); katarzyna.ciesielczyk@put.poznan.pl (K.C.); mateusz.fabisiak@put.poznan.pl (M.F.)

**Keywords:** cold-formed thin-walled profiles, point load, strengthening, point crimping, experiment, numerical modelling, Eurocode 3

## Abstract

The article provides information about strengthening cold-formed thin-walled steel beams made of the sigma profile. An innovative concept for sectional transverse strengthening of thin-walled beams subjected to concentrated forces was investigated. The proposed solution’s novelty lies in attaching the sectional transverse strengthening to the beam’s cross-section, employing a point crimping technique. This technique requires a specific modification of the cross-section edges, necessitating double-lipped flanges. This strengthening method is innovative, as it has not been previously applied to cold-formed structures. Typically, strengthening is achieved using other cold-formed elements or materials, such as timber, lightweight concrete, or CFRP tapes. The laboratory experimentally validated the proposed method using short- and medium-length beams. The experimental results were then compared with the results of the numerical analyses and the conventional design approach described in EC3. The results demonstrated the feasibility of implementing this type of strengthening, its reliability under load, and the confirmation of an increase in the load-bearing capacity of the experimental samples by 11–24%.

## 1. Introduction

Cold-formed thin-walled steel structures are commonly used as load-bearing structures for roofs or walls, specifically as purlins or sheeting. At the same time, beams made from cold-formed thin-walled (TWCF) profiles are in demand, particularly during reconstructions or construction projects involving complex logistical challenges. In such conditions, the low weight of TWCF elements, combined with a wide variety of cross-sections, allows for the solution of many engineering tasks [1,2,3]. Considering the above, it can be observed that the use of lightweight thin-walled structures results in low environmental costs, expressed through reduced material consumption and lower transportation and assembly costs. Consequently, these structures align with the principles of sustainable construction, which aim to reduce CO_2_ emissions. However, it is worth noting that using TWCF structures is not the only method to reduce the carbon footprint of steel structures. Research and initiatives focused on the steel production process are also noteworthy. For example, one approach to mitigating environmental impacts involves the implementation of carbon capture and utilization (CCU) solutions in steel production. The CCU concept entails the chemical binding of CO_2_ into valuable chemicals. In a study [4], the environmental impacts of BF-BOF steel plants with and without CCU were compared based on life cycle assessment analyses (LCA). An alternative approach to reducing environmental impacts is the adoption of steel–wood hybrid structures. Studies [5,6] investigate the potential applications of hybrid wood–steel beams under static and dynamic loading, respectively. Additionally, high-strength steel is another effective method for lowering the carbon footprint. Research [7] analyses the mechanical properties of S600E steel, outlining its prospective applications in engineering structures.

Previous research by various investigators has noted that TWCF elements are susceptible to locally applied loads [8,9,10,11,12]. This characteristic of such structures raises the issue of strengthening the points where loads are applied or where the elements rest on other structures. This study is dedicated to addressing this problem.

One method of increasing the load-bearing capacity of TWCF elements is due to advances in modern manufacturing technologies, which allow profiles to be produced with varying cross-sections. These advances optimize global-local buckling behaviour [13] and enhance ultimate strength by exploiting the post-buckling response of beams subjected to local distortional buckling modes [14]. In such cases, the load-bearing capacity of cold-formed elements depends on the cross-sectional shape, thickness, and grade of steel used in beams [15] and columns [16,17]; this is because the configuration of the cross-section (e.g., edge bends, indentations on the web and flanges) significantly impacts the element’s load-bearing capacity. Currently, selecting an optimal cross-sectional shape is one of the primary strategies for reducing steel consumption in the production of cold-formed thin-walled structures, which fits in with sustainable building guidelines [18,19,20,21].

A promising method for enhancing the load-bearing capacity of thin-walled cold-formed (TWCF) elements involves strengthening steel structures with carbon fibre-reinforced polymer (CFRP) strips, as suggested in several studies [22,23,24]. However, manufacturers of thin-walled structures have not yet adopted this technique. It remains in the research phase and is primarily employed in custom solutions for reinforcing existing structures. Aware of studies dedicated to strengthening local sections of beams to enhance resistance to concentrated force [25]. Such methods are more “traditional” and involve additional fastening elements, increasing the structure’s labour intensity.

The literature highlights alternative methods for reinforcing TWCF structures. Studies [26,27] present an innovative technique for strengthening TWCF elements through an original bolt-and-nut system, investigated using experimental and numerical analyses. This method significantly enhances compression strength [26] and bending strength [27]. The effectiveness of the proposed approach was validated through comprehensive experimental and numerical assessments. Another method of reinforcing TWCF structures involves connecting open sections, such as channel or sigma cross-sections, in a back-to-back configuration. The study of [28] examines a new generation of back-to-back cold-formed steel (CFS) channels featuring edge-stiffened holes. An alternative concept for reinforcing TWCF structures involves filling closed sections with a concrete mixture. This reinforcement method targets compression members, such as columns [29,30].

This paper addresses the strengthening of channel and sigma profiles, typically manufactured through cold rolling and cold bending processes, by adding sectional transverse strengthening connected to the beam’s cross-section using the point crimping method. The point crimping method (PCM) of joining the sectional transverse strengthening with the TWCF element is original, as it has not yet been used in building structural applications. The discussed strengthening method (PCM) allows for unrestricted placement of transverse strengthening along the length of the element. Such an element can be used as a floor beam, roof purlin, wall girder, or platform beam in industrial structures. There is no such solution in the literature concerning construction structures. An alternative method to increase the load-bearing capacity of a thin-walled element is, for instance, optimizing its cross-sectional shape. For example, patent description no. P.403898 [31] proposes bending the web inward (similar to sigma-shaped sections) and adequately shaping the flanges. Another approach involves strengthening by adhesively bonding CFRP strips, as described in utility model no. W.130147 [32]. Additionally, patent description no. P.354157 [33] and P.372028 [34] refer to methods for manufacturing thin-walled sections. The patent [33] (P.354157) proposes connecting two flanges with vertical bridges clamped onto the flanges. The other patent [34] (P.372028) describes connecting two flanges with diagonal ribs arranged in an X-shape, with the ribs clamped onto the flanges. Both solutions describe the connection of two independent strips, resulting in a double-branched cross-section (whereas the proposed invention involves single-branched elements).

The study will present the point crimping method (PCM) application for connecting sectional transverse strengthening (STS) with beam elements. However, the proposed solution can be easily extended to constructing entire systems, such as grids or transverse frames, where components are joined using appropriately bent plates attached to sections with PCM.

A key advantage of the point crimping method is the elimination of commonly used joining techniques, such as welding and bolting, in the manufacturing and assembly of thin-walled structures. Welding is typically avoided in thin-walled structures due to the small thickness of the sheet metal and the damage to the anticorrosion coating during the welding process.

Moreover, using hot-dip galvanized MAGNELIS^®^ sheets (with a zinc coating alloyed with aluminium and magnesium) significantly enhances anticorrosion properties and is well-suited for laser cutting. Production methods involving laser cutting and press brakes provide substantial design flexibility and enable the elimination of costly and inefficient processes such as welding, shot blasting, painting, and the transportation of oversized components.

## 2. Materials and Methods

**Figure 1:**
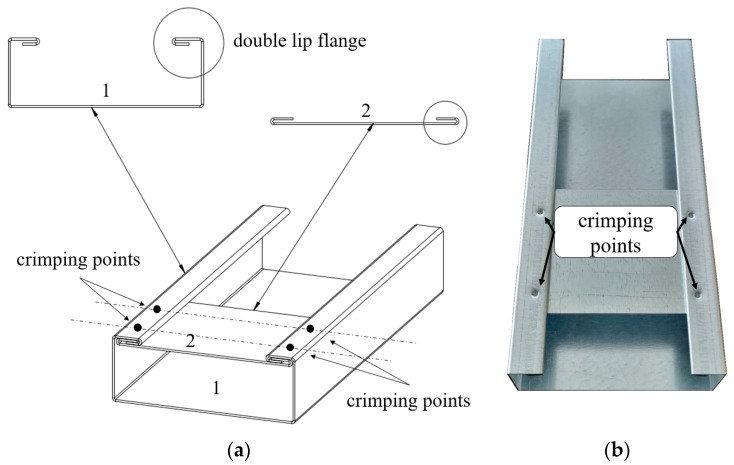
Point crimping method (PCM) for strengthening cold-formed thin-walled elements with double-lip flanges: (**a**) schematic idea, where 1—TWCF element, 2—sectional transverse strengthening—STS, black dots represent crimping points; (**b**) realization.

The concept of strengthening cold-formed thin-walled structures using sectional transverse strengthening (STS) joined by the point crimping method (PCM), see Figure 1, arising from the collaboration between researchers at Poznan University of Technology and ARPSTAL Antoni Ratajczak company (Poznań, Poland).

**Figure 2:**
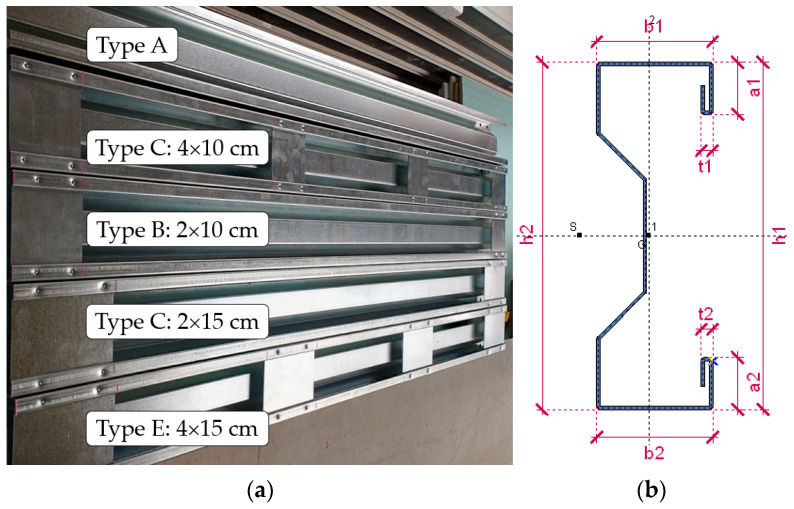
Tested TWCF sigma beams: (**a**) variants of the STS; (**b**) parametrization of cross-section.

The PCM method enables the creation of a cross-section with a double lip on the flanges, allowing a sectional transverse strengthening element (in the manuscript, the STS abbreviation will also be interchangeably used) to be inserted into the gap created by the lip. The strengthening element is a sheet of the same material as the main beam. This sheet has two bends at its ends, connecting it to the double lips of the investigated profile flanges. This solution enables the enhancement of the load-bearing capacity of a cross-section by locally closing it, which, on the one hand, increases its resistance to concentrated forces and, on the other, locally improves its torsional strength. The innovation of this solution lies in the fact that local strengthening is performed without commonly used joining methods such as welding or bolting. To the authors’ knowledge, no cases of using crimping to join structural elements in construction have been reported thus far. This method can be classified as a type of press joint. A custom device for sheet clamping was developed to implement the concept of PCM. The device is designed to enable point-specific crimping by forming an indentation in the double-lip flange of the cross-section and crimping it together with the double-lip of the sectional transverse strengthening. The rosette joint is the most well-known press joint widely used in the production of cold-formed steel structures [35]. A total of 60 sigma-section beam samples were produced. The samples consisted of beams of two lengths: 160 cm (short span beam L_1_ = 150 cm, S150) and 210 cm (long span beam L_2_ = 200 cm, S200). These beams exhibit varying slenderness in the context of lateral–torsional buckling (this is the only form considered due to the absence of axial compressive force). Such slenderness is determined as the ratio of the buckling length (in our case, 150 cm and 200 cm divided by i_0_ = (i_y_^2^ + i_z_^2^)^0.5^ = (5.73442 + 1.76572)^0.5^ = 6.0 cm hence, short and long beams are characterized by corresponding slenderness ratios λ_LT,1_ = 150/6.0 = 25.0 and λ_LT,2_ = 200/6.0 = 33.3. Each beam length was produced in 5 variants (6 specimens for each variant); see Table 1 and Figure 2a.

Figure 2b depicts the sigma cross-section geometry with an indication of the inventory control segments. The beams and transverse sectional strengthening were made of a hot dip galvanized sheet (nominal thickness 1.5 mm), MAGNELIS^®^, coated with the addition of aluminium and magnesium, which significantly improves its anticorrosion properties and is ideal for laser cutting processing [36,37]. Due to its properties, this coating provides long-term surface protection. The chemical composition of the coating ensures optimal anticorrosion properties. The coating consists of zinc alloys (93.5%), aluminium (3.5%) and magnesium (3%).

Table 2 shows the results of the cross-section dimension inventory. The measurements were taken using an electronic caliper with a resolution of 0.01 mm and an accuracy of ±0.02 mm for measurements up to 100 mm and ±0.03 mm for measurements above 100 mm. The average wall thickness of the section and transverse sectional strengthening is 1.48 mm ± 0.03 mm.

**Figure 3:**
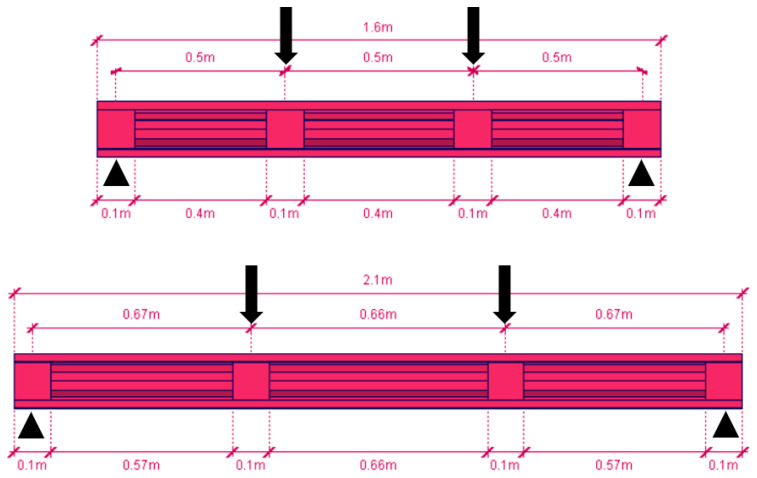
Static scheme for four-point bending tests—visualization of short (S150) and long (S200) beams with four transverse section strengthening of a length 10 cm each.

Two lengths of beams were planned for tests in a four-point bending scheme. Figure 3 shows the support spacing and load application points for both beam lengths. The span for the shorter beam was 150 cm, while for the longer beam, it was 200 cm.

### 2.1. Testbed Description

**Figure 4:**
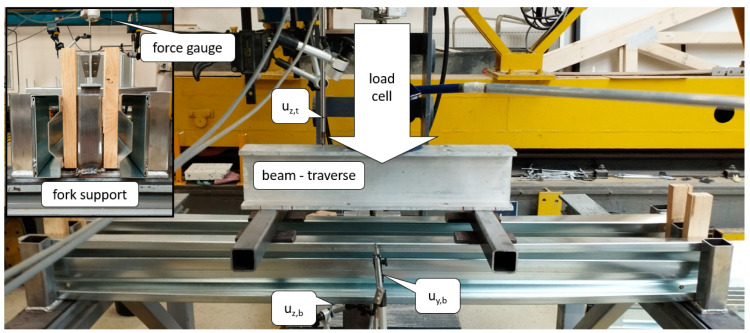
Four-point bending—view of the testbed (description in the text).

In the study, washers were used to increase the load transfer area to avoid stress concentrations at the support and applied load points. The practical support length was 70 mm at the support and 133 mm at the span. To enforce symmetrical load transfer throughout the load range, it was decided to test a system of two beams symmetrically aligned with respect to the machine axis. Figure 4 depicts a view of the test bed, which allows four-point bending tests.

Figure 4 also presents the support (fork support), the position of the force gauge (50 kN class 0.5, which gives a force reading accuracy of ±0.25 kN) and the five displacement transducers (one 1-WA/100MM-T and four 1-WA/50MM-T transducers with a linearity deviation of ±0.2%) used during the test namely: vertical displacement measurement of the traverse—u_z,t_, vertical displacement of the bottom flange for the both beams—u_z,b_, horizontal displacement of the lip of the top flange for both beams—u_y,b_. The registration of displacement and force was carried out at a frequency of 5 Hz.

### 2.2. Material Properties of Galvanized Sheet MAGNELIS

**Figure 5:**
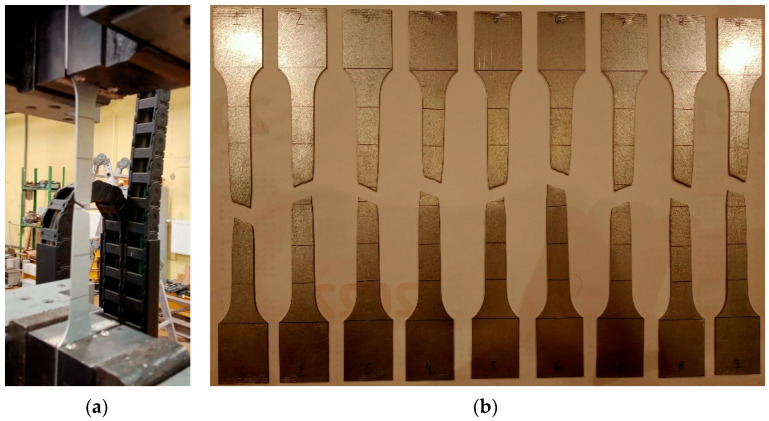
Static tensile test: (**a**) view of a sample in the testing machine; (**b**) specimens following the static tensile test.

Material tests were carried out to determine the steel’s yield strength and tensile strength used in the experiments. Material testing was performed by ISO standard [38] using an Instron Satec testing machine (Norwood, MA, USA) with a maximum capacity of 300 kN. The uniaxial tensile tests were carried out on nine rectangular specimens with gripped ends cut from the same plate from which the thin-walled beams were made. The gauge base of the prepared specimens was equal to 20 × 160 mm. The tensile test of the steel was divided into two phases related to the tensile rate of the specimen. In the first stage, the test rate was controlled by the rate of stress increase according to method B [38]. An initial stress rate of 2 MPa/s was applied to a nominal specimen strain of 0.2% (elastic range). In this part of the test, the strain value of the specimen was measured using an extensometer with a base length of 50 mm. When the specimen strain reached 0.2%, the extensometer was removed, and further measurements were taken directly from the Instron testing machine. At this stage (plastic region), the tensile rate of the specimen was controlled at a strain rate of 0.35 mm/s—method A [38]. Figure 5 presents specimens following the static tensile test. All specimens failed correctly, i.e., their fracture did not occur near the grips of the testing machine. The results of the static tensile test of the steel are summarised in Table 3.

**Figure 6:**
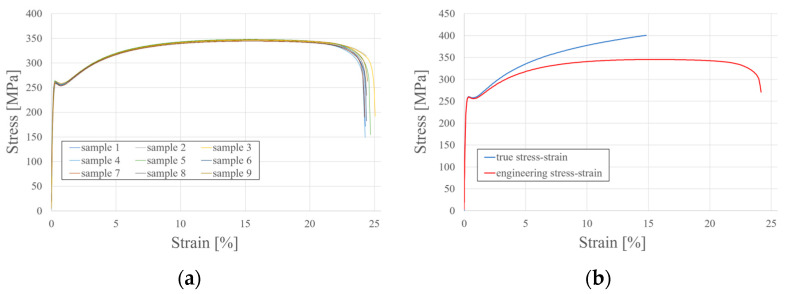
Stress–strain relationship: (**a**) 9 samples from static tensile test; (**b**) true stress–strain relationship for numerical investigation in Abaqus CAE.

The stress–strain diagram is depicted in Figure 6a. For the specimens tested, the average yield strength equals f_y_ = 261.0 MPa, and the tensile strength equals f_u_ = 345.6 MPa. The above material tests were also used to define the materials in the numerical model in the Abaqus environment. It is worth noting that the curve developed from the laboratory tests represents the engineering stress–strain relationship, where the following formulas define stress and strain:

(1) $$\sigma_{nom}=\frac{P}{A_{0}},$$

(2) $$\varepsilon_{nom}=\frac{∆l}{l_{0}},$$

where *P* is the axial tensile force, *A*_0_ is the initial cross-sectional area of the specimen, Δ*l* is the elongation of the specimen, and *l*_0_ is its initial length.

However, when defining the plastic model of the material in the Abaqus/CAE 2019 program, the actual values of stresses and strains [39,40] defined by Equations (3)–(5) must be used. In the calculations, the value of Young’s modulus was assumed, according to European standard [41], to be 210 GPa.

(3) $$\sigma_{true}=\sigma_{nom}\left(1+\varepsilon_{nom}\right),$$

(4) $$\varepsilon_{true}=ln\left(1+\varepsilon_{nom}\right),$$

(5) $$\varepsilon_{true}^{pl}=\varepsilon_{true}-\frac{\sigma_{true}}{E},$$

where *σ_true_* and *ε_true_* are the actual stresses and strains, and $\varepsilon_{true}^{pl}$ is the true plastic strain. Using the above relationships, a curve representing the relationship between the actual stresses and strains was defined and contrasted with the mean curve representing the relationship between their engineering equivalents (Figure 6b).

## 3. Results

### 3.1. Experimental Approach

Laboratory results for S150 and S200 TWCF beams are summarized in Table 4 and Table 5, respectively. In Table 4 and Table 5, the beam configurations are represented as follows:

- A represents beams without STS;
- B represents beams with two ten cm-long STS located at the supports;
- C represents beams with four ten cm-long STS, positioned at the supports and midspan;
- D represents beams with two 15 cm-long STS located at the supports;
- E represents beams with four 15 cm-long STS positioned at the supports and midspan.

Additionally, in Table 4 and Table 5, the vertical displacement of the bottom flange of the individual beams is represented by u_z,b_, the vertical displacement of the traverse beam is represented by u_z,t_, and the F_t,max_ represents the maximal force applied to the traverse beam (with standard deviation). In contrast, a change of the F_t,max_ is referred to as a beam without STS.

**Figure 7:**
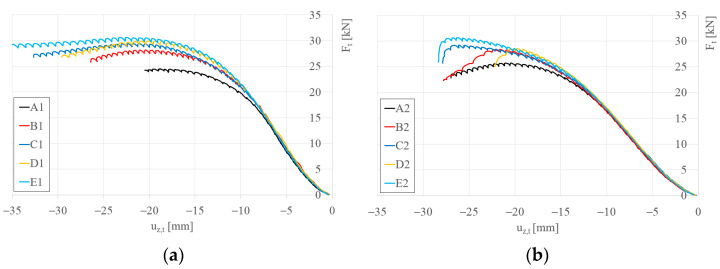
Load–vertical displacement of traverse beam (u_z,t_) equilibrium paths: (**a**) S150 beams; (**b**) S200 beams.

Figure 7 presents the load–displacement curves for the analyzed beams, demonstrating that the application of sectional transverse strengthening (STS) increases the load-bearing capacity of the S150 and S200 beams. Three ranges can be identified for all recorded equilibrium paths. The first range shows nonlinear characteristics, the second is linear, and the third range becomes nonlinear again. The nonlinearity in the initial range is associated with the clearance adjustment of the testing system. The nonlinearity in the third range arises from the activation of beam failure mechanisms.

**Figure 8:**
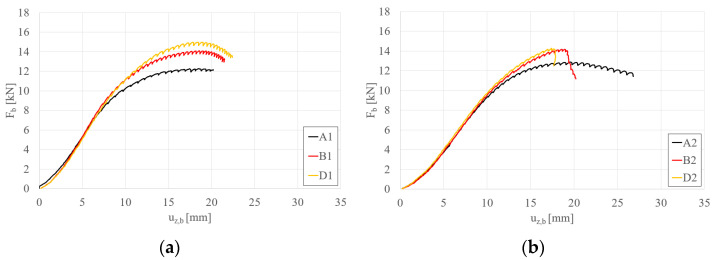
Load (F_b_)–vertical displacement (u_z,b_) equilibrium paths: (**a**) S150 beams; (**b**) S200 beams—STS at the support.

**Figure 9:**
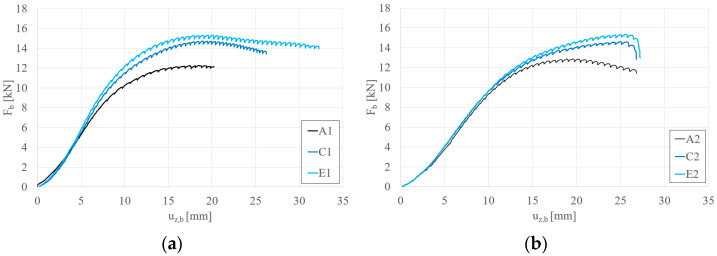
Load (F_b_)–vertical displacement (u_z,b_) equilibrium paths: (**a**) S150 beams; (**b**) S200 beams—STS at the support and in the middle.

**Figure 10:**
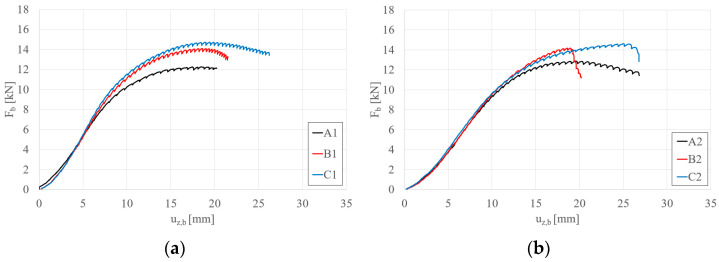
Load (F_b_)–vertical displacement (u_z,b_) equilibrium paths: (**a**) S150 beams; (**b**) S200 beams—STS 10 cm long at the support vs. STS 10 cm long at the support and in the middle.

**Figure 11:**
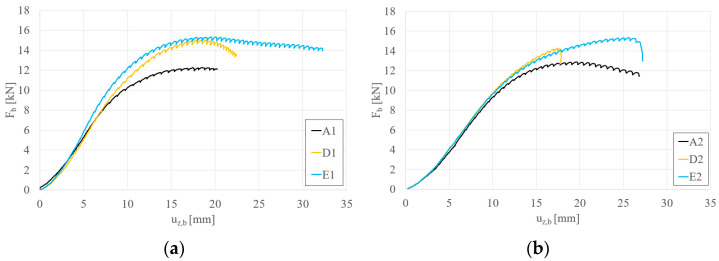
Load (F_b_)–vertical displacement (u_z,b_) equilibrium paths: (**a**) S150 beams; (**b**) S200 beams—STS 15 cm long at the support vs. STS 15 cm long at the support and in the middle.

Detailed comparisons of the effects of STS are illustrated in Figure 8, Figure 9, Figure 10 and Figure 11. Please note that the force per beam Fb is half of the force applied to the system F_t_. Figure 8 illustrates the effect of strengthening lengths of 10 cm and 15 cm applied in the support zone. It can be observed that adding STS in the support zone effectively increases the maximum load capacity of the beam. The effect for short beams S150 (Figure 8a) is slightly more significant than for longer beams S200 (Figure 8b). Furthermore, the difference between STS lengths of 10 cm and 15 cm is noticeable in the case of short beams; this is because, for longer beams, the global stability of the tested beams starts to play a role. The influence of STS in the support zone can be expressed by the parameter β, which represents the ratio of F_b,STS_ (load capacity of the beam with STS reinforcement) to F_b,N_ (load capacity of the beam without STS reinforcement). For the graphs in Figure 8, the value of β equals 1.15, 1.21, 1.11, and 1.13 for beams B1, D1, B2, and D2, respectively.

Figure 9a,b present the influence of 10 cm and 15 cm reinforcements applied to the span and support zones for beams S150 and S200, respectively. Similarly to the case of reinforcements applied only in the support zone (types B and D), the effect of additional reinforcements in the span zone is more pronounced for shorter beams (S150, Figure 9a). Furthermore, it was observed that the failure mode of beams with four reinforcements (regardless of their length) for longer beams (S200, Figure 9b) resulted in a sudden loss of load-bearing capacity (global instability combined with crushing at the support). Using the introduced parameter β, its C1, E1, C2, and E2 values are 1.19, 1.24, 1.13, and 1.19, respectively.

Figure 10a,b depict the effect of two and four reinforcements, each 10 cm long, on beams S150 and S200, respectively. For both beam lengths, an increase in load-bearing capacity was observed with the application of four reinforcements. However, the increase in capacity is marginal, with β rising from 1.15 to 1.19 for beams S150 and 1.11 to 1.13 for beams S200.

Figure 11a,b depict the effect of two and four reinforcements, each 15 cm long, on beams S150 and S200, respectively. For both beam lengths, an increase in load-bearing capacity was observed with the application of four reinforcements. However, the increase in capacity is marginal, with β rising from 1.21 to 1.24 for beams S150 and from 1.13 to 1.19 for beams S200.

### 3.2. Failure Mechanisms

**Figure 12:**
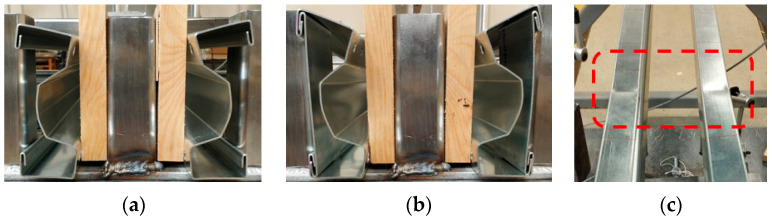
The failure mechanism of short-span beams (S150): (**a**) at the support without STS; (**b**) at the support with STS; (**c**) indentation (marked in red box) under the applied force in the middle (beams C1 and E1).

For short beams spanning 150 cm (S150), failure at the support (refer to Figure 12) was the dominant failure mode, irrespective of the beam variant. However, the load levels at which failure occurred varied, as illustrated in the graphs in Figure 7. In this failure scenario, the load-bearing capacity is governed by the ability to transfer the support reactions. This failure mechanism is characterized by significant deformability, resulting in a gradual rather than sudden failure, which extends over time. From the perspective of structural reliability, this type of failure is considered favourable. As shown in Figure 12b, the STS connection to the double-lipped edge did not fail, and it is evident that the STS limited the “opening” of the section in the support zone, thus increasing the load-bearing capacity of the tested beams. Additionally, indentation was observed in the upper flange under applied force in the case of beams with four STSs, namely beams C1 and E1 (Figure 12c). No lateral torsional deformation was observed in the short beams.

**Figure 13:**
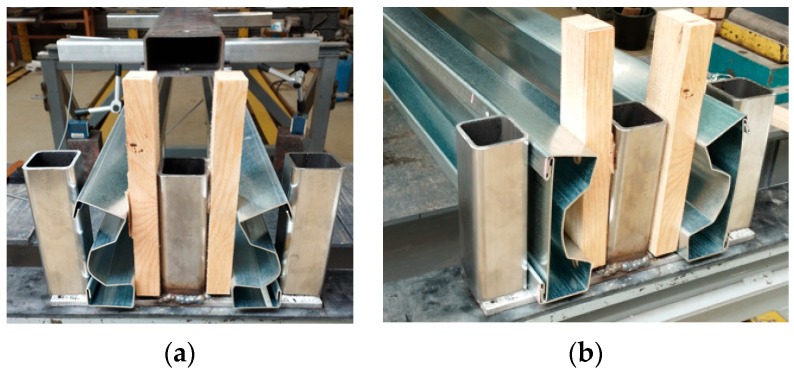
Failure mechanisms of long-span beams (S200) at the support: (**a**) without STS; (**b**) with STS.

For long-span beams with a 200 cm span (S200), failure was observed at the supports, similar to the S150 beams (see Figure 12 and Figure 13). Furthermore, in the case of S200 beams without STS reinforcement (type A2), this failure mode was the only one observed (see Figure 13a), and its intensity was significantly more significant compared to the S200 beams with STS (see Figure 13b); this resulted in a force–displacement equilibrium path exhibiting the highest ductility, comparable to that of S150 beams.

**Figure 14:**
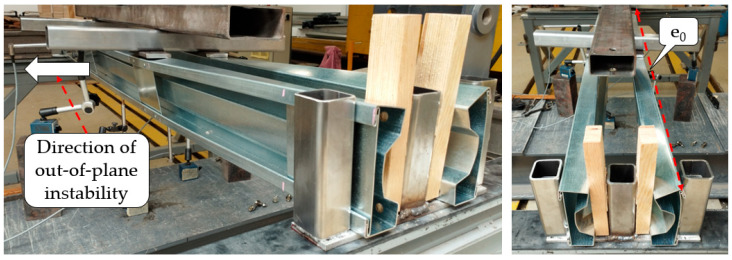
Out-of-plane failure mechanisms (lateral–torsional deformation) of long-span beams (S200).

In beams with sectional transverse strengthening (STS)—specifically, beams B2, C2, D2, and E2—torsional deformation was observed in the span, indicating lateral–torsional buckling (see Figure 14). This phenomenon involves the beam “exiting” the plane of bending, resulting in an increased eccentricity between the load plane and the shear centre of the cross-section. Consequently, the torsional moment acting on the cross-section increases. The sudden collapse of the equilibrium paths for these beams suggests a loss of global stability due to lateral–torsional buckling. Notably, in unreinforced beams, no out-of-plane deformation was observed, with failure occurring solely at the supports.

### 3.3. Analytical Approach

**Figure 15:**
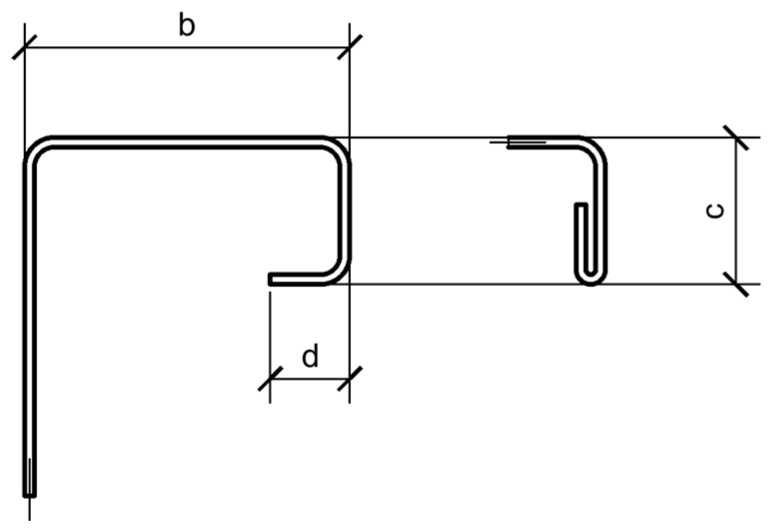
Cross-section with a double flange lip, according to [43].

This part presents the assessment of the compliance of specimens with the requirements of EN 1993-1-3 [42,43]. Based on the test results, it was established that the yield strength of the steel is f_yb_ = 261 MPa, the ultimate strength is f_u_ = 345.6  MPa, and Young’s modulus is E = 210 GPa. The measured mean value of sheet thickness was used in analytical calculation, namely t = 1.48 mm. The cross-section of the studied beams has the following geometric parameters: h/t = 150/1.48 = 101.35 < 500; b/t = 50/1.48 = 33.8 < 90; c/t = 22.5/1.48 = 15.2 < 60; d/t = 12/1.48 = 8.1 < 50. Two additional conditions must be satisfied for cross-sections with flange lips: 0.2 ≤ c/b = 22.5/50 = 0.45 ≤ 0.6; 0.1 ≤ d/b = 12/50 = 0.24 ≤ 0.3. Thus, this cross-section complies with the requirements of Clause 5.2 [42] and Clause 7.4 [43], and its resistance to various internal forces can be evaluated according to these standards. It is worth noting that the Eurocode allows for the arrangement of a double lip, where section “c” is parallel to section “d” (see Figure 15). However, the calculation method only suggests a variant where section “d” is perpendicular to section “c”.

**Figure 16:**
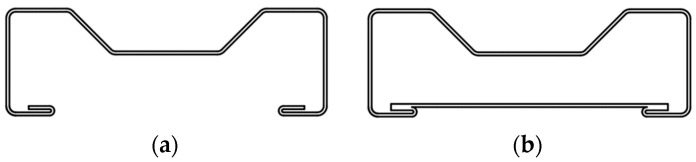
Sample cross-sections: (**a**) without strengthening; (**b**) with vertical strengthening.

Therefore, in the calculations, we will consider two variants: one with a double lip and one with a single lip. The results of both calculations indicate no influence of local buckling or cross-sectional stability when bending, as W_el_ = W_eff_. This means that the section parameters should be used for strength calculations. Given this, we will attempt to evaluate the load-bearing capacity of the two cross-sections shown in Figure 16. The cross-section in Figure 16a represents the cross-section of the studied beams with a double lip, where both lips are parallel. The cross-section in Figure 16b represents a cross-section with an additional strengthening element. Since the press-fit connection of the plate to the sigma profile has not been investigated in this study, the plate will be conditionally assumed to be connected to the stiffening elements of the sigma profile flanges.

The calculation results of the cross-sectional resistances are presented in Table 6. Since the sample cross-section is effective under bending, the bending strength assessment used the average yield strength f_ya_. The difference in values for the two cross-sections is due to differences in their area. To determine the shear resistance of the cross-sections, the web of the sigma profile and the flat web of the strengthening element were conditionally separated. V_b,Rd1_—shear resistance of the sigma profile web, V_b,Rd2_—shear resistance of the plate-like strengthening element, V_b,Rd_—total shear resistance of the cross-section.

The most relevant strength criterion is the resistance to shear forces (referred to as local transverse forces in the 2006 edition). To determine the resistance of the sigma profile web, a reduction factor k_st,w_ must be applied. At the same time, for webs with longitudinal stiffening elements, the requirement of (6.21) [42] or (8.49) [43] must be met. The position of the furthest point of the stiffening element from the line connecting the ends of the web is 20 mm. Thus, e_w,max_/t = 20/1.48 = 13.5 exceeds 12 and does not meet the previous two requirements.

Different editions of the Eurocode provide different methodologies for assessing resistance to local transverse forces. Given that during the tests, the beams were braced at the supports with wooden blocks, the resistance at the support was evaluated according to formula (6.16a) [42]. In Table 6, this result is denoted as R_w,Rd1_, and R_w,Rd1_red_ refers to the local force resistance of the sigma profile web. The resistance of the unreinforced section (R_w,Rd2_) was also determined. The methodology for calculating shear force resistance, according to [43], has undergone changes. The results of the beam’s resistance to the support reaction were calculated using formula (8.33) [43], for a section of type “2” according to Figure 8.6 [43], which is not restrained at the support. This resistance is denoted in Table 2 as R_w,Rd3_. We will transition from the load-bearing capacity based on various parameters presented in Table 6 to the corresponding load values applied through the testing press to compare the theoretical results with the experimental and numerical studies. The calculated force values are presented in Table 7. The values of the ultimate forces on a single beam will be halved.

The nature of deformations at the supports (see Figure 10 and Figure 11) indicates that full restraint of the beam web from rotation does not occur. Accordingly, it can be concluded that the ultimate strength values of the web under the influence of shear forces R_w,Rd1_ and R_w,Rd1_red_ should be excluded from the analysis. The following method can be proposed for the theoretical assessment of the strength of a beam with sectional transverse strengthening (beam with STS) under the action of shear forces. Let us assume that the resistance of the beam web corresponds to the resistance values R_w,Rd2_red_ or R_w,Rd3_red_ when calculated according to the first and second-generation Eurocodes, respectively. In this case, the resistance of the reinforcing element will correspond to the resistance values R_w,Rd2_ and R_w,Rd3_. Since the beam is not reinforced along its entire length, we can apply the hypothesis that the reinforcing element will resist a shear force proportional to the total length of the reinforcement elements in the beam. We denote this proportionality coefficient as k_ls_:

(6) $$k_{ls}=l_{s}/l_{b},$$

where *l_s_* is the total length of the vertical reinforcing elements, and *l_b_* is the geometric length of the beam. Thus, the total resistance *R_w,Rd,tot_* of the reinforced section (in beams with STS) to shear forces at the support can be expressed as:

(7) $$R_{w,Rd,tot}=R_{w,Rd\_red}+k_{ls}R_{w,Rd},$$

where *R_w,Rd_red_* is the section’s resistance with a web containing longitudinal stiffening elements, and *R_w,Rd_* is the section’s resistance without longitudinal stiffening elements. The ultimate shear resistances of the web, calculated using Formula (2), are presented in Table 8.

**Figure 17:**
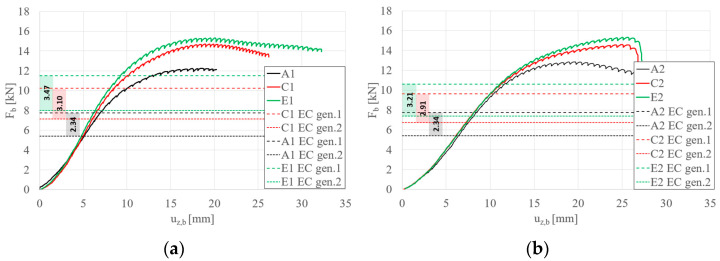
Diagram only for example and discussion: (**a**) beam S150; (**b**) S200.

Since the forces listed in Table 8 correspond to the force Fb shown in Figure 8 and Figure 9, Figure 17 graphically represents these values along selected equilibrium path plots. The graphs also highlight the differences in load-bearing capacities determined according to Eurocode 3 gen. 1 and 2. The capacities calculated using EC3 gen. 2 fall within the linear range of the equilibrium paths obtained from the tests, making them significantly more conservative. In this case, the EC3 gen. 2 formulas provide a more accurate definition of shear capacity.

### 3.4. Numerical Approach

**Figure 18:**
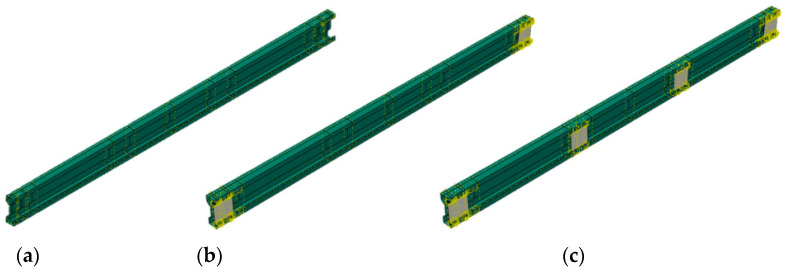
Assembly of numerical model in Abaqus environment: (**a**) beam without STS; (**b**) beam with two STS; (**c**) beam with four STS.

The finite element (FE) model (see Figure 18) was developed using the Abaqus/CAE environment, employing the Newton–Raphson (static general) solution procedure. Abaqus software is often used to simulate and solve engineering problems and to support experimental research in building engineering applications [44,45]. Nonlinear geometric effects were incorporated into the Abaqus FE model, ensuring that the analysis accounted for nonlinear shape changes and deformations that could influence the calculation outcomes. This approach allows for a comprehensive evaluation of the nonlinear behaviour of the elements under investigation. The true stress–strain relation (Figure 6b) was also implemented in the material definition, introducing the material nonlinearity in this way. In summary, the finite element simulations were solved using the geometrically and materially nonlinear analysis (GMNA).

**Figure 19:**
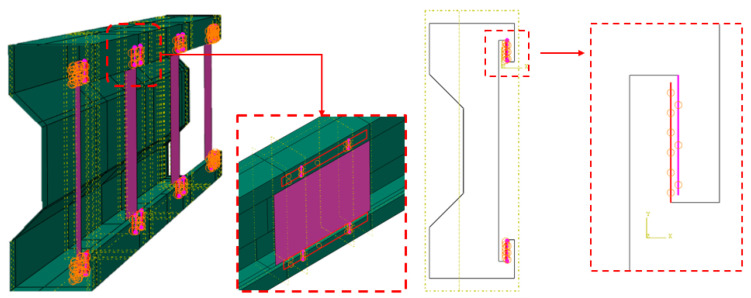
Tie connection visualization in assembly model: surface to surface.

The STS components were connected to the beam element via a tie constraint (see Figure 19). In Abaqus, a tie connection is a constraint that allows two separate surfaces to behave as if they are perfectly bonded. This type of connection can be used when different model parts need to act as a single unit, even though they may be meshed separately. The tie constraint enforces continuity of displacements and rotations across the surfaces, effectively preventing separation or sliding.

**Figure 20:**
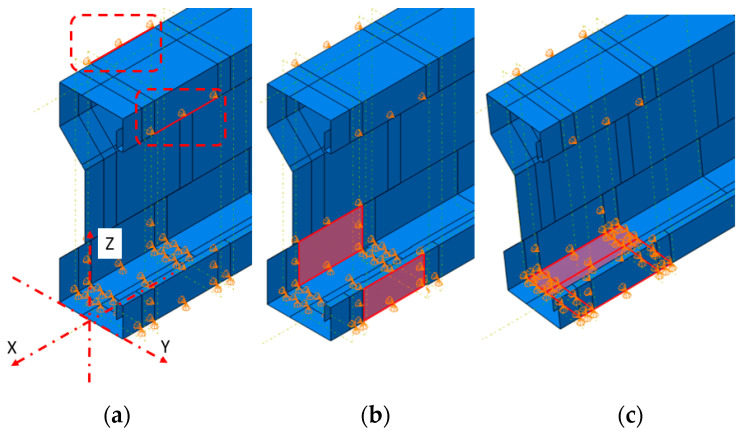
Boundary conditions: (**a**) line BC—u_Y_ = 0.0; (**b**) surface BC—u_Y_ = 0.0; (**c**) surface BC—u_Y_ = 0.0 and u_Z_ = 0.0.

In the numerical model, two vertical displacement/rotation-type surface supports were defined, along with four lateral displacement/rotation-type surface supports applied to the lower and upper flange of the beam, see Figure 20. Such defined support conditions correspond to pin fork supports employed during the laboratory experiments.

**Figure 21:**
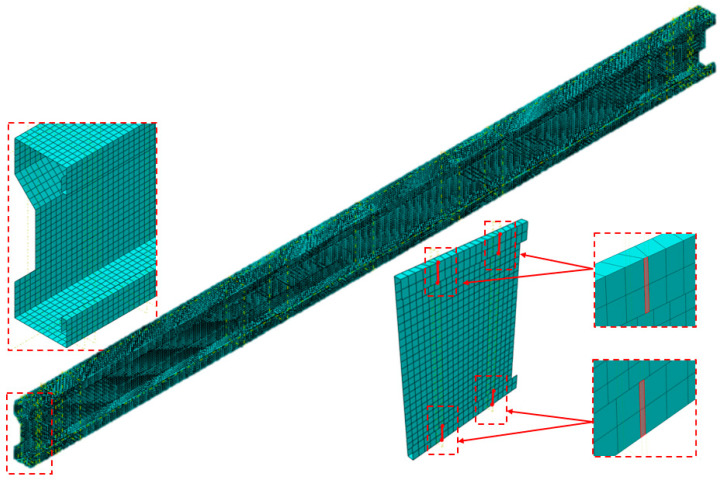
Meshing of the numerical model.

The steel elements (cold-formed Σ-beam with or without sectional transverse strengthening) were modelled using four-node double-curved thick shell elements with reduced integration (type S4R). A mesh size of 5 mm was used; see Figure 21.

The loading process was simulated in three steps. In the first step, the self-weight of the beam was modelled as the applied load. This load was evenly divided, with one portion applied across the entire surface of the top flange and the other across the bottom flange, thus accurately representing the self-weight of the tested elements within the FE model. The second step simulated the loading from the testing apparatus, which was modelled in Abaqus to mirror the experimental conditions. This included the self-weight of the devices used for preliminary loading, applied at the exact location as the final beam load. In the third step, the final experimental load was applied as a surface load of 5 N/mm² at two specific points on the beam.

**Figure 22:**
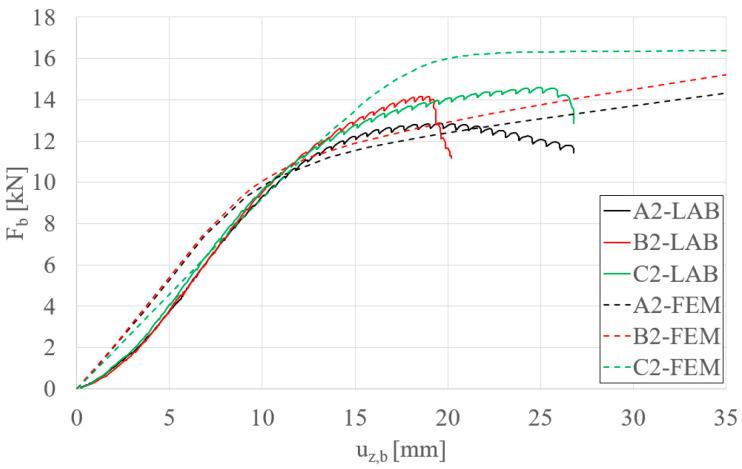
Load–displacement plots—comparison of FEM results with laboratory experimental data.

Figure 22 presents the results for S200 beams, corresponding to the selected beam variants: A2 (without STS), B2 (with two STS elements of 10 cm length), and C2 (with four STS elements of 10 cm length). Continuous lines represent laboratory experiment data, while dashed lines correspond to finite element (FE) analysis results. Overall, there is satisfactory convergence between the numerical and experimental models. However, the FE model for the beam without STS closely matches the laboratory results. For beams with two and four STS elements, the FE model underestimates and overestimates the experimental data, respectively.

Numerical analyses were conducted on a personal laptop with the following specifications: 32 GB RAM, AMD Ryzen 7 4800H processor (2.90 GHz, eight cores/16 threads), NVIDIA GeForce RTX 2060 graphics card, and an M.2 SSD. The analysis time for a single task was ~5000 s.

## 4. Discussion

The impact of the STS elements on increasing the load-bearing capacity of cold-formed thin-walled sigma profile members falls within the range of 15–24% for short S150 beams (Table 9) and between 11 and 19% for long S200 beams (Table 10). The obtained results indicate that strengthening at the support already yields a significant increase in load-bearing capacity, although it is higher for short beams than for long ones. This is because, in the case of short beams, the shear capacity of the element plays a more important role, whereas, for long beams, bending becomes more influential. Adding reinforcement in the central zone (at the point of load application) further increases the load-bearing capacity of the element by a few per cent for both short and long beams.

## 5. Conclusions

Experimental and numerical studies confirmed the effectiveness of the proposed strengthening method for flexural cold-formed steel sigma section elements. The load-bearing capacity of the strengthened beams increased by 11–24% compared to beams without strengthening. These results were achieved through the following measures:

(a) compliance with standards:
  – verify that the proposed designs comply with current standards (first and second generations of Eurocodes);
  – determination of the resistance of the samples using analytical normative methods;
  – development of a methodology for assessing the resistance to shear forces at the support for strengthened beams;
(b) experimental research:
  – load-bearing capacity tests on sigma beams with two spans (150 cm and 200 cm),
  – implementation of various strengthening schemes at supports and points of concentrated load application;
(c) numerical simulation:
  – a numerical experiment using the ABAQUS software package to replicate laboratory tests.

The study demonstrated the effectiveness of the proposed strengthening methods. The suggested strengthening connection method proved successful during laboratory testing, ensuring the full attachment of the strengthening elements to the beam across the entire load range. The proposed numerical model accurately replicates the mechanical response of beams without strengthening and satisfactorily represents beams with strengthening.

It should be noted that the study was conducted for beams with asymmetric, open cross-sections relative to the vertical axis, which imposed certain limitations on the results’ applicability.

The findings suggest the feasibility of applying this method in engineering practice. Furthermore, the proposed strengthening approach opens additional research opportunities. For example, it is necessary to investigate the impact of this strengthening on the load-bearing capacity of compressed elements made from single C- and sigma profiles, with particular interest in studying cross-sectional shape instability. Additionally, further investigation is required to understand the behaviour of double-fold edge stiffeners and the effects of local instability and cross-sectional shape stability on the performance and structural integrity of this cross-sectional form.

## Figures and Tables

**Table 1:** List of cold-formed thin-walled beam variants.

Type	No. of STS ^1^	Length of STS	STS at the Ends	STS in the Middle
A	0	−	−	−
B	2	10 cm	yes	−
C	4	10 cm	yes	yes
D	2	15 cm	yes	−
E	4	15 cm	yes	yes

^1^ STS—sectional transverse strengthening.

**Table 2:** Cross-section dimension inventory results (example for two samples).

Sample	h_1_ [mm]	h_2_ [mm]	a_1_ [mm]	a_2_ [mm]	b_1_ [mm]	b_2_ [mm]	t_1_ [mm]	t_2_ [mm]
	149.28	149.38	22.62	22.50	50.32	50.36	5.25	5.16
1	149.22	149.43	22.67	22.64	50.36	50.40	5.26	5.15
	149.29	149.51	22.67	22.71	50.36	50.40	5.24	5.18
	149.77	149.91	22.68	22.76	50.40	50.46	5.14	5.23
2	149.91	149.86	22.72	22.81	50.41	50.43	5.13	5.22
	150.06	149.93	22.69	22.70	50.47	50.39	5.17	5.21
m.v.	149.6		22.7		50.4		5.2	
s.d.	0.3		0.1		0.0		0.0	

**Table 3:** Data from static tensile test.

Samples	1	2	3	4	5	6	7	8	9	m.v.	s.d.
f_y_ [MPa]	262.20	260.67	260.73	260.80	263.62	258.89	258.50	261.76	261.81	261.00	1.60
f_u_ [MPa]	347.30	344.70	346.10	344.30	347.90	344.30	344.20	344.90	347.10	345.60	1.48

**Table 4:** The results of the four-point bending of a beam span of 150 cm (please refer to Table 1, Figure 2).

Measured Parameter	A1	B1	C1	D1	E1
F_t,max_ [kN]	24.70 ± 0.23	28.50 ± 0.39	29.40 ± 0.01	29.80 ± 0.07	30.60 ± 0.04
change of F_t,max_ with respect to A1 test [%]	−	15	19	21	24
u_z,t_ [mm]	18.60 ± 0.20	20.70 ± 0.31	21.70 ± 0.52	20.60 ± 0.33	22.20 ± 0.31
u_z,b_ [mm]	18.03 ± 0.31	18.64 ± 0.22	18.90 ± 0.32	17.99 ± 0.60	18.90 + 0.32

**Table 5:** The results of the four-point bending of a beam span of 200 cm (please refer to Table 1, Figure 2).

Measured Parameter	A2	B2	C2	D2	E2
F_t,max_ [kN]	25.60 ± 0.12	28.40 ± 0.08	29.00 ± 0.19	28.80 ± 0.30	30.50 ± 0.15
change of F_t,max_ with respect to A2 test [%]	−	11	13	13	19
u_z,t_ [mm]	21.20 ± 0.29	21.80 ± 0.19	24.60 ± 1.46	20.20 ± 0.40	24.90 ± 1.90
u_z,b_ [mm]	20.33 ± 0.82	20.18 ± 1.06	23.10 ± 1.99	18.88 ± 0.97	23.69 + 1.57

**Table 6:** Calculation results according to [42,43].

Parameter	Type of Cross-Section I (Figure 16a)	Type of Cross-Section II (Figure 16b)
Area, mm^2^	478	669
Moment of inertia, mm^4^	1,558,243	1,823,001
Basic yield strength f_yb_, N/mm^2^	261	261
Ultimate tensile strength f_u_, N/mm^2^	345.6	345.6
Average yield strength f_ya_, N/mm^2^	280.39	274.85
Elastic section modulus W_el_, mm^3^	20,777	24,307
Plastic section modulus W_pl_, mm^3^	22,743	26,829
Bending moment resistance M_c,Rd_, kNm	5.62	6.56
Shear resistance V_b,Rd1_, kN	33.27	33.27
Shear resistance V_b,Rd2_, kN	−	22.5
Shear resistance V_b,Rd_, kN	33.27	55.77
Coeff. for calc. the resis. of a stiffened web to transverse force k_s,tw_	0.774	−
Resistance of the web to transverse force by [42]		
R_w,Rd1_, kN	9.95	−
R_w,Rd1_red_, kN	7.70	−
R_w,Rd2_, kN	5.00	−
R_w,Rd2_red_, kN	3.87	−
Resistance of the web to transverse force by [43]		−
R_w,Rd3_, kN	3.49	−
R_w,Rd3_red_, kN	2.70	−

**Table 7:** Values of the applied ultimate forces [kN] for different strength conditions of the cross-section.

Parameter	S150 (Figure 16a)	S200 (Figure 16a)	S150 (Figure 16b)	S200 (Figure 16b)
Ultimate force for bending moment resistance	44.96	33.72	52.48	39.36
Ultimate force for shear resistance	133.08	133.08	223.08	223.08
Ultimate force for resistance of the web to transverse force for R_w,Rd1_	39.78			
for R_w,Rd1_red_	30.85			
for R_w,Rd2_	20.00			
for R_w,Rd2_red_	15.48			
for R_w,Rd3_	13.95			
for R_w,Rd3_red_	10.80			

**Table 8:** The ultimate shear resistance of the web of beams with STS.

Beam S150	*R_w,Rd,tot_* (acc. EC3 1-gen)	*R_w,Rd,tot_* (acc. EC3 2-gen)	Beam S200	*R_w,Rd,tot_* (acc. EC3 1-gen)	*R_w,Rd,tot_* (acc. EC3 2-gen)
A1	7.74	5.40	A2	7.74	5.40
B1	8.99	6.27	B2	8.69	6.06
C1	10.24	7.14	C2	9.64	6.73
D1	9.62	6.71	D2	9.17	6.40
E1	11.49	8.02	E2	10.60	7.39

**Table 9:** Comparison of load-bearing capacities for S150 beams.

Beam S150	A1	B1	C1	D1	E1
F_max_ [kN]	12.4	14.3 (+15%)	14.7 (+19%)	14.9 (21%)	15.3 (+24%)

**Table 10:** Comparison of load-bearing capacities for S200 beams.

Beam S200	A2	B2	C2	D2	E2
F_max_ [kN]	12.8	14.2 (+11%)	14.5 (+13%)	14.4 (13%)	15.2 (+19%)

## Data Availability

The original contributions presented in this study are included in the article. Further inquiries can be directed to the corresponding author.
